# Health Tract, No. 71

**Published:** 1865

**Authors:** 


### HEALTH TRACT, No. 71.

### NERVOUS SUFFERERS.

There are a multitude of advertisements of books and remedies in reference to "nervous debility," as it is called. For a purpose, we once sent a few stamps for an infallible receipt for consumption or some other ailment. The answer came that it was the "Indian Plant, growing at the foot of the Rocky Mountains; and that it had to be gathered in August, otherwise it had no virtues." As it would cost something to go to the Rocky Mountains, and it was then December; and in addition, no description was given of the "Indian Plant," and as there might not be any Indians at the foot of the Rocky Mountains, after we got there, to point out the "Indian Plant," we were in a quandary; but the whole set of almost insuperable difficulties were vanquished in a moment by the announcement that the advertiser had some of it on hand at two dollars a bottle. The *American Agriculturist* of New-York, conducted with so much energy and ability by Orange Judd, for a dollar a year, each monthly number containing not less than one hundred separate articles, all practical and useful, thus exposes one of the various forms of impositions:

"'Rev. Wilson' a Humbug.—We supposed we had exposed the person advertising over this name sufficiently to put all readers of the *Agriculturist*, at least, on their guard. But our newer readers may need a new caution, at least we so judge from several recent letters of inquiry. It may therefore be said in brief, that we have never yet been able to find 'Rev. Wilson.' He formerly claimed to have been a preacher in the 'New-Haven Conference.' This falsehood we nailed by reminding our readers that Methodists themselves never heard of any 'New-Haven Conference.' He now substitutes the 'New-England Conference.' His own advertisements in the *New-York Tribune* and *Times* he quotes in his pamphlet as if they were editorial indorsements from those papers. (They deserve this for even inserting his advertisements.) This so-called Rev. Wilson (like all the rest of the class) pretends to great benevolence, and a desire to benefit the afflicted without charge. He sends a 'prescription' it is true, but while by specious statements he almost, or quite convinces the nervous suffering that his medicine will be their salvation, he is very careful to inform them that the medicine is very costly and difficult to get in a pure state, and that the safest way is to send him (the 'Rev. Wilson') *two dollars*, and fifteen cents more for postage, and get the genuine medicine. This is the practice of all these 'retired physicians,' retired clergymen, and other benevolent humbugs. That they deceive many, is evident, as they continue to expend very large sums in advertising."

It should be remembered that there is only one remedy for the class of ailments referred to—rest for the parts implicated—that is abstinence, temperance, and a building-up of the general health; not by tonics, but by a plain diet and a constant employment in out-door activities. Another heartless swindle and imposition in this direction is, that the very state of things which proves the vigor of the system is artfully presented as a symptom of decline. See "Sleep." By Dr. W. W. Hall, New-York.

